# Trends in the utilization of acupuncture among children in Taiwan from 2002 to 2011: a nationwide population-based study

**DOI:** 10.1186/s12906-019-2753-8

**Published:** 2019-11-21

**Authors:** Chieh Wang, Yu-Chen Lee, Mei-Yao Wu, Cheng-Li Lin, Mao-Feng Sun, Jaung-Geng Lin, Hung-Rong Yen

**Affiliations:** 1 Department of Chinese Medicine, China Medical University Hospital, Taichung, 404 Taiwan; 2 Graduate Institute of Acupuncture Science, College of Chinese Medicine, China Medical University, Taichung, 404 Taiwan; 3 School of Post-baccalaureate Chinese Medicine, College of Chinese Medicine, China Medical University, Taichung, 404 Taiwan; 4 Management Office for Health Data, China Medical University Hospital, Taichung, 404 Taiwan; 5 School of Chinese Medicine, College of Chinese Medicine, China Medical University, 91 Hsueh-Shih Rd, North District, Taichung, 404 Taiwan; 6 Research Center for Traditional Chinese Medicine, Department of Medical Research, China Medical University Hospital, Taichung, 404 Taiwan; 7 Research Center for Chinese Herbal Medicine, China Medical University, Taichung, 404 Taiwan; 8 Chinese Medicine Research Center, China Medical University, Taichung, 404 Taiwan; 9 Department of Biotechnology, Asia University, Taichung, 413 Taiwan

**Keywords:** Acupuncture, Children, Complementary and alternative medicine, Epidemiology, National Health Insurance Research Database, Taiwan

## Abstract

**Background:**

In recent years, acupuncture has been increasingly integrated into pediatric care worldwide. However, recent epidemiological studies about pediatric users of acupuncture are lacking. The current study aimed to fill the gap and carry out the large-scale investigation on the basis of the pediatric population in Taiwan.

**Methods:**

We conducted a nationwide population-based study to investigate the utilization of acupuncture in Taiwan. We analyzed data from the Longitudinal Health Insurance Database 2000 (LHID 2000). The datasets contained all original claims data for 1 million beneficiaries who were randomly sampled from the registry of all beneficiaries enrolled in the Taiwan’s National Health Insurance Program from January 1, 2000 to December 31, 2011. Children younger than 18 years old were enrolled into our study for analysis. The demographic data, treatment modalities and distributions by disease categories of the pediatric acupuncture users were analyzed by descriptive statistics. Logistic regression analysis was used to investigate the trends in acupuncture use over time.

**Results:**

The one-year prevalence of pediatric acupuncture users increased from 1.78% in 2002 to 5.34% in 2011. Acupuncture use significantly increased each year (*p*-value< 0.0001). Patients who were male, of greater age, resided in highly urbanized areas and suffered from injury or disorders of the musculoskeletal system were more likely to accept acupuncture treatment. Infantile cerebral palsy and psychoses were the top two health issues among those receiving complex acupuncture treatment. Older (> 9 years old) children tended to receive acupuncture treatment due to injury and musculoskeletal system disorders more than younger (≤9 years old) children.

**Conclusions:**

Our study revealed that the utilization of acupuncture in pediatrics became increasingly popular year by year in Taiwan from 2002 to 2011. The results of this study may provide some valuable information for further clinical practice and acupuncture research, as well as to the government and societies concerning pediatric health care.

## Introduction

Complementary and alternative medicine (CAM) has been popular among patients worldwide for several decades [1–3]. According to a US survey conducted in 2007, approximately 38% of adults and 12% of children had previously used several types of CAM therapy, with a total of USD 33.9 billion out-of-pocket spending [4, 5]. In UK pediatric populations, the average lifetime prevalence rate of CAM-use was 42%, which was disclosed by a survey in 2013 [6]. Of the different types of CAM, traditional Chinese medicine (TCM) has commonly been used by not only the Chinese population but also other ethnic groups [7].

One of the most popular forms of TCM [8], acupuncture, has been practiced in China for more than 3000 years. Acupuncture is a form of treatment modality which involves inserting thin needles through the skin at specific points on the body. It is believed to regulate the flow of Qi through the meridians in order to correct the imbalance of energy in the body and restore natural internal homeostasis [9]. Due to the utility in treating a variety of conditions, such as stroke [10–12], pain-related diseases [13, 14], anxiety and depression [15–18], acupuncture has also been growing in prominence in many parts of the world [19–24]. Acupuncture has been generally practiced in Taiwan as well not only in local clinics but also medical centers. Based on the nationwide population-based study in Taiwan, acupuncture use among adults significantly increased each year from 7.98% in 2002 to 10.9% in 2011 [25].

Acupuncture has been increasingly used in pediatric care for a variety of health issues including asthma [26], cerebral palsy [27, 28], complications of cancer [29, 30], etc. On the basis of a 2005 US survey, 33% of pediatric pain centers in America offered acupuncture as a part of their services [31]. Based on evidence from several previous reviews, the efficacy of acupuncture in children for certain conditions is encouraging [8], such as in postoperative symptoms, chemotherapy-induced nausea/vomiting and pain [32]. Acupuncture also seems to be quite well-tolerated in children and has been applied to the pediatric population with low incidence of adverse effects [32, 33].

To date, very few of studies have examined the trends in acupuncture use, especially within the pediatric population. A few previous studies in Taiwan have placed importance on the usage of acupuncture among adults [20, 25]. However, there is a lack of large-scale, population-based, epidemiological analyses regarding the utilization of acupuncture in children. Previous literature focused on either the whole age range of acupuncture users or specific diseases with a limited sample size. The comprehensive demographics and pattern studies of pediatric acupuncture users were rarely reported. In order to understand the trends in acupuncture use within the pediatric population in Taiwan, we conducted this nationwide population-based study to investigate the utilization of acupuncture in children from the real-world data in Taiwan.

## Materials and methods

### Data source

Taiwan established the National Health Insurance (NHI) program in 1995. One year later, TCM service was also reimbursed by the NHI program in addition to the western medical service. As one part of TCM services, acupuncture treatment including manual acupuncture, electroacupuncture and complex acupuncture have also been practiced for insured patients. Besides, the program covered more than 99% of the total population in Taiwan [34]. All claims data, including de-identified demographic information (e.g., sex, birth dates, occupation and place of residence) and clinical information (e.g., diagnostic codes based on the International Classification of Disease, 9th Revision, Clinical Modification [ICD-9-CM], health management and treatment), has been collected in the large computerized NHI research database (NHIRD). This nationwide database is highly reliable [35], which provides researchers with information to evaluate the utilization of TCM.

In this study, we used the Longitudinal Health Insurance Database 2000 (LHID 2000) as the source of datasets from the National Health Research Institutes in Taiwan. The LHID 2000 consists of the original claims data for 1 million beneficiaries who were randomly sampled from the registry of all beneficiaries enrolled in the NHI program in 2000 and followed longitudinally until 2011. The registry comprises the beneficiary characteristics, such as age, geographic region and place of residence, which are updated every year. Other de-identified information regarding the use of medical care facilities, specialties, sex, birth dates, visit dates, prescriptions, health management, costs and diagnostic codes based on the International Classification of Disease, 9th Revision, Clinical Modification (ICD-9-CM) [36] were all included in the datasets.

### Study samples

We analyzed data regarding acupuncture users less than 18 years old by the treatment codes defined by the NHI, including manual acupuncture (B41, B42, B80-B84, B90-B94, P27041, P31103, P32103 and P33031), electroacupuncture (B43, B44, B86–89 and P33032) and complex acupuncture (B45 and B46). “Complex acupuncture” is defined by the NHI as an acupuncture treatment for patients with catastrophic illness (e.g., cerebral vascular disease, cancer, infantile cerebral palsy and psychiatric disorders) or epilepsy [25].

### Study variables

In our study, we analyzed independent variables such as sex, age, level of urbanization and geographic region to explore the effects on acupuncture utilization. Children younger than 18 years old were enrolled into the study of the 1 million beneficiaries. To investigate the differences among the various age groups, the sampled pediatric enrollees were sequentially categorized into the following 6 subgroups in accordance with age: ≤3, 4 to 6, 7 to 9, 10 to 12, 13 to 15 and > 15 years old (y/o).

The total population is approximately 23 million in Taiwan. Residence areas were also grouped into 4 levels of urbanization depending on the population density (people/km^2^), the ratio of elderly persons, the ratio of people with different educational levels, the ratio of agricultural workers and the number of physicians per 100,000 persons [37]. The highest degree of urbanization was level 1, and the lowest was level 4.

### Statistical analysis

The data were analyzed by SAS statistical software (version 9.4 for Windows; SAS Institute, Inc., Cary, NC, USA). The descriptive statistics of the demographic data, treatment modalities and distributions by disease categories of the pediatric acupuncture users were also collected and organized. The percentage of acupuncture users by different demographic factors such as gender, age, level of urbanization and geographic region was calculated as the number of acupuncture users divided by the sampled enrollees by different demographic factors. We also conducted the logistic regression for trend test to investigate the change in the utilization rate of acupuncture over time.

### Ethical consideration

The NHIRD was provided by the National Health Insurance Administration and managed by the National Health Research Institutes of Taiwan. All of the datasets were de-identified and encrypted to protect enrollees’ privacy; there was no possibility to identify individual patients from them. This study was approved by the Research Ethics Committee of China Medical University and Hospital (CMUH104-REC2–115). The patient consent was exempted for the total anonymity of all research data in this study.

## Results

Children under 18 years old randomly sampled as part of the 1 million beneficiaries of the LHID 2000 from 2002 to 2011, defined as “valid beneficiaries” were included in our study. The total numbers of valid beneficiaries were 218,820 in 2002 and 95,361 in 2011, with 3900 and 5088 pediatric acupuncture users respectively (Table 1). Our data revealed that 40,219 pediatric patients in total underwent acupuncture treatment from 2002 to 2011. The prevalence of pediatric acupuncture users was significantly higher from 1.78% in 2002 to 5.34% in 2011 (*p*-value< 0.0001). Female acupuncture users increased from 1.51% in 2002 to 4.52% in 2011, whereas male acupuncture users increased from 2.03% in 2002 to 6.09% in 2011. The proportion of male acupuncture users increased significantly than female after adjusting the years (*p*-value< 0.0001). New subjects in Table 1 indicate the new pediatric patients who start to received acupuncture treatment for each year. The numbers of new subjects were 2912 in 2002 and 2933 in 2011 respectively. Acupuncture users of the different urbanized residence areas all increased from 2002 to 2011. Residents in higher urbanization areas were more likely to receive acupuncture.

**Table 1:** Number of pediatric patients among acupuncture users from 2002 to 2011 in Taiwan

Year	Valid beneficiaries within NHI^a^	Subjects with acupuncture use							
		Total numbers (%)^b^	New subjects (%)^c^	Male (%)^d^	Female (%)^d^	Urbanization▵			
						level 1 (%)	level 2 (%)	level 3 (%)	level 4 (%)
2002	218,820	3900 (1.78)	2912 (74.7)	2311 (2.03)	1589 (1.51)	59,534 (2.02)	64,816 (1.68)	43,397 (1.84)	51,073 (1.57)
2003	203,557	3878 (1.91)	2652 (68.4)	2272 (2.15)	1606 (1.64)	55,292 (2.14)	60,334 (1.76)	40,362 (1.89)	47,569 (1.82)
2004	189,706	3967 (2.09)	2624 (66.1)	2401 (2.43)	1566 (1.72)	51,405 (2.26)	56,334 (2.11)	37,617 (2.05)	44,350 (1.90)
2005	176,813	3699 (2.09)	2271 (61.4)	2225 (2.41)	1474 (1.74)	47,901 (2.22)	52,499 (2.16)	35,089 (2.05)	41,324 (1.89)
2006	163,601	3411 (2.08)	2026 (59.4)	2052 (2.41)	1359 (1.73)	44,399 (2.27)	48,492 (2.11)	32,466 (2.09)	38,244 (1.85)
2007	149,957	3401 (2.27)	2046 (60.2)	2040 (2.61)	1361 (1.90)	40,640 (2.57)	44,415 (2.29)	29,789 (2.08)	35,113 (2.05)
2008	136,213	3542 (2.60)	2108 (59.5)	2203 (3.10)	1339 (2.05)	36,905 (2.89)	40,261 (2.69)	27,072 (2.47)	31,975 (2.26)
2009	122,450	4080 (3.33)	2499 (61.3)	2467 (3.87)	1613 (2.75)	33,195 (3.69)	36,181 (3.52)	24,283 (3.25)	28,791 (2.76)
2010	109,152	5253 (4.81)	3389 (64.5)	3114 (5.49)	2139 (4.08)	29,525 (5.76)	32,244 (4.90)	21,670 (4.70)	25,713 (3.60)
2011	95,361	5088 (5.34)	2933 (57.6)	3019 (6.09)	2069 (4.52)	25,832 (6.83)	28,118 (5.46)	18,961 (4.93)	22,450 (3.80)
2002–2011		40,219	25,460	24,104	16,115	12,427	12,011	7764	8017

^a^Valid beneficiaries within NHI refer to the pediatric patients randomly sampled as part of the 1 million beneficiaries from the LHID 2000. ^b^Total numbers of acupuncture users indicate total acupuncture users of the sampled pediatric enrollees. ^c^New subjects indicate the new pediatric patients who start to received acupuncture treatment for each year, while the percentage refers to the new acupuncture users out of total numbers of acupuncture users each year. ^d^% of male/female acupuncture users describes the percentage of male/female acupuncture users out of all male/female beneficiaries (data not shown) each year. ▵% of acupuncture users in the different urbanization levels means the percentage of acupuncture users out of all beneficiaries in each urbanized area (data not shown)

According to the NHI Administration, there were 6 regional divisions in Taiwan, including Taipei and the Northern, Middle, Southern, Kao-Ping, and Eastern divisions. We also found that the percentages of pediatric acupuncture users were highest in the Taipei branch bureau and lowest in the Southern branch bureau (data not shown).

Table 2 demonstrates acupuncture use with age during the 10 years. Children older than 15 years old made up the majority of acupuncture users. Approximately 5.04% of them received acupuncture treatment, followed by the 13–15 y/o and 10–12 y/o groups. Children under 3 years old had the least number of visits compared to the other age groups, with only 30 (0.12%) of them undergoing acupuncture from 2002 to 2011.

**Table 2:** Age-specific cumulative prevalence of acupuncture use during 10 years from 2002 to 2011 in Taiwan

Age (y/o)	Number of total population^a^	Number of acupuncture users (%)^b^	Number of acupuncture visits^c^
> 15	400,396	20,193 (5.04%)	36,609
13–15	398,514	12,181 (3.06%)	21,604
10–12	363,172	6104 (1.68%)	11,237
7–9	249,163	1415 (0.57%)	3264
4–6	129,287	296 (0.23%)	1060
≤3	25,108	30 (0.12%)	109
Total	1,565,640	40,219	73,883

^a^Number of total population in 6 subgroups indicates the amount of the different age groups pediatric patients randomly sampled as part of the 1 million beneficiaries from the LHID 2000 during 10 years

^b^Number of acupuncture users in 6 subgroups refers to the amount of different age groups pediatric acupuncture users, while the percentage of each age group describes the acupuncture users out of numbers of sampled pediatric enrollees during 10 years

^c^Number of acupuncture visits means the total number of acupuncture visits in different age groups during 10 years

Pediatric patients who received acupuncture treatment used manual acupuncture most frequently (84.9%) (Table 3). Electroacupuncture was used in a minority (5.2%) of the children, followed by the complex acupuncture group.

**Table 3:** Cumulative frequency distribution of acupuncture users in each treatment modality from 2002 to 2011 in Taiwan

Treatment modalities	Treatment codes	^a^*N* = 40,219 (%)
Acupuncture	B42	34,140 (84.9)
Acupuncture + Herbal Medicine	B41	3954 (9.83)
Electroacupuncture	B44	1778 (4.42)
Electroacupuncture + Herbal Medicine	B43	312 (0.78)
Complex Acupuncture	B45	2 (0.00)
Complex Acupuncture + Herbal Medicine	B46	33 (0.08)

^a^N: numbers of acupuncture users in each treatment modality

Pediatric patients suffering from infantile cerebral palsy (ICD-9 code: 343) and psychoses with origin specific to childhood (ICD-9 code: 299) were the top two health issues among those receiving complex acupuncture (Table 4). The percentages were 46.1 and 37.1%, respectively.

**Table 4:** The most common diseases of patients who received complex acupuncture cumulated from 2002 to 2011 in Taiwan

Diseases	ICD-9	^a^*N* = 3648 (%)
Infantile cerebral palsy	343	1680 (46.1)
Psychoses with origin specific to childhood	299	1352 (37.1)
Epilepsy	345	260 (7.13)
Subarachnoid hemorrhage	430	202 (5.54)
Intracerebral hemorrhage	431–432	132 (3.62)
Cerebral infarction	433–434	14 (0.38)
Myasthenia gravis	358	6 (0.16)
Other diseases of spinal cord	336	2 (0.05)

^a^N: frequency of total complex acupuncture use in different disease categories during 10 years

Table 5 provides the different disease categories that received acupuncture treatment in pediatric patients from 2002 to 2011 in Taiwan. Injury and poisoning (ICD-9 code: 800–999) was the largest population (67.0%), whether in age group ≤9 or > 9 y/o (Table 5). In the age group > 9 y/o, diseases of the musculoskeletal system and connective tissue (ICD-9 code: 710–739) were the second most common reason leading to acupuncture visits (23.9%). In addition, diseases of the nervous system and sense organs (ICD-9 code: 320–389) (19.4%) and mental disorders (ICD-9 code: 290–319) (19.4%) were the second and third most common health issues that children received acupuncture treatment in the ≤9 y/o group, respectively.

**Table 5:** Cumulative frequency distribution of pediatric acupuncture users by disease categories and age groups from 2002 to 2011 in Taiwan

Diagnosis (ICD-9-CM range)	Acupuncture N (%)^a^	Age (year)		
		≤9 (%)	> 9 (%)	*p*-value
Injury and Poisoning (800–999)	375,887 (67.0)	13,342 (35.0)	362,545 (69.4)	< 0.001
Diseases of the Musculoskeletal System and Connective Tissue (710–739)	130,525 (23.3)	5417 (19.4)	125,108 (23.9)	< 0.001
Diseases of the Nervous System and Sense Organs (320–389)	20,438 (3.64)	7411 (19.4)	13,027 (2.49)	< 0.001
Mental Disorders (290–319)	13,198 (2.35)	7394 (14.2)	6457 (1.24)	< 0.001
Diseases of the Respiratory System (460–519)	8584 (1.53)	2127 (5.58)	5804 (1.11)	< 0.001
Symptoms, Signs, and Ill-Defined Conditions Injury and Poisoning (800–999)	5494 (0.98)	1661 (4.36)	3833 (0.73)	< 0.001
Diseases of the Digestive System (520–579)	2306 (0.41)	384 (1.01)	1922 (0.37)	< 0.001
Diseases of the Skin and Subcutaneous Tissue (680–709)	1876 (0.33)	170 (0.45)	1706 (0.33)	< 0.001
Diseases of the Genitourinary System (580–629)	1000 (0.18)	2 (0.42)	998 (0.19)	0.78
Diseases of the Circulatory System (390–459)	863 (0.15)	162 (0.07)	701 (0.13)	< 0.001
Endocrine, Nutritional, and Metabolic Diseases, and Immunity Disorders (240–279)	363 (0.06)	26 (0.06)	337 (0.06)	0.30
Neoplasms (140–239)	181 (0.03)	21 (0.01)	160 (0.03)	0.01
Infectious and Parasitic Diseases (001–139)	115 (0.02)	5 (0.01)	110 (0.02)	–
Complications of Pregnancy, Childbirth, and the Puerperium (630–676)	1 (0.00)	1 (0.00)	0 (0.00)	< 0.001
Diseases of the Blood and Blood-Forming Organs (280–289)	0 (0.00)	0 (0.00)	0 (0.00)	< 0.001

^a^N: frequency of acupuncture use among pediatric patients in different disease categories during 10 years

## Discussion

Our study found that the prevalence of pediatric acupuncture users in Taiwan has substantially increased from 2002 to 2011. Males had a higher acceptance rate (calculated by dividing the number of male/female acupuncture users by the number of total male/female beneficiaries each year) than females. A majority of the acupuncture users in children were older than 15 years old. Residents in higher urbanization areas were more likely to receive acupuncture. Of the six regions, the percentage of one-year acupuncture usage was highest in Taipei branch bureau. Injury was the major category most commonly treated with acupuncture in pediatric patients. Patients suffering from infantile cerebral palsy and psychoses with origin specific to childhood were the largest population of those receiving complex acupuncture.

Overall, we provided some useful information not only for health care but also for further epidemiological research. The importance of this study was based on several aspects. For example, on the basis of the literature review, there was a lack of research regarding the trends in use of acupuncture among children in western countries and even in Chinese society. Most previous studies of the utilization of acupuncture focused on either the whole age range of acupuncture users or specific diseases with a limited sample size. The complete demographics and pattern studies of pediatric acupuncture users were rarely reported; our study appears to be the first large-scale and population-based investigation of this issue. Moreover, we assessed the real-world data from the NHI program for use as our datasets so the potential for selection bias could be minimized.

In recent years, not only TCM but also acupuncture have increasingly been integrated into pediatric health care in parts of the world [8]. According to the 2007 National Health Interview Survey, approximately 150,000 children under 18 years old had used acupuncture in the past year [38]. Our results were also consistent with the previous study focused on TCM users in Taiwan, with the rate of pediatric acupuncture usage increasing among TCM users from 2005 to 2010 [30]. Compared to the former study, our data samples were from the total population instead of TCM users, which might provide the overall status of utilization of acupuncture among children in Taiwan. Although acupuncture has become gradually accepted in the pediatric population in Taiwan, the acceptance of it was still lower than other therapeutic approaches of TCM, such as herbal remedies and manipulative therapies [30]. This might be due to several reasons. For example, acupuncture has been perceived to be a type of invasive treatment. Parents whose children have needle phobia, poor immune function or bleeding diseases were more likely to choose the other conservative managements of TCM, including herbal medicine, manipulative therapies and even laser acupuncture treatment [39, 40]. Furthermore, laser acupuncture, which is a novel, noninvasive and painless short-term therapy, is currently a self-paid treatment in Taiwan, which means that it was excluded from the LHID 2000, as well as from our datasets.

We found a male predominance among pediatric acupuncture users, which was different from previous reports among adults in Taiwan [25]. Based on the results that injury was the major health issue for receiving acupuncture treatment in pediatric patients, this might in part account for the male predominance. Previous studies have found that boys were more likely to experience most kinds of injuries and be involved in behaviors that were highly correlated with injury [41]. Therefore, they had higher injury risks due to the psychosocial and environmental influences during infancy and these rates increased throughout childhood [42]. For the age group > 9 y/o, disorders of the musculoskeletal system and connective tissue were the second most common reason leading to acupuncture visits; therefore, it might be speculated that due to their participation in athletic activities, injuries and musculoskeletal disorders, such as sprains or contusions, occurred more frequently [30]. According to TCM theory based on Yellow Emperor’s Inner Canon (so-called Huangdi Neijing), these disorders result in Qi stagnation and blood stasis in terms of TCM diagnoses. Acupuncture is considered as the strategy in order to relieve the symptoms by moving qi and quickening the blood [43].

Interestingly, children suffering from mental and neurological diseases made up a portion of acupuncture users for the age group ≤9 y/o. One reason for this could be related to developmental retardation or developmental disability. The early intervention service (EIS) for children with developmental delay has been a booming welfare issue in Taiwan in recent years [44]. Acupuncture for diseases such as cerebral palsy [27, 45] and autism spectrum disorder (ASD) [46] plus rehabilitation training have been widely used for children with developmental delay due to the therapeutic effectiveness. In children with cerebral palsy, acupuncture plus rehabilitation training improved gross motor function, reduced muscle spasms, and enhanced daily life activities [45]. Several reviews also suggested that acupuncture may be an effective treatment for alleviating the symptomatology of ASD [46, 47]. Acupuncture also showed some promise for other mental and neurological diseases, such as anxiety [48], nocturnal enuresis [49] and visual impairment [50, 51]. However, there was weak evidence to support this use of acupuncture due to limited information.

Similarly, our study revealed that children older than 15 y/o made up the majority of acupuncture users, followed by the 13–15 y/o and 10–12 y/o groups. Children under 3 years old had the least number of visits compared to the other age groups. Those findings were consistent with previous reports on pediatric CAM use in the United States (16.4% for children aged 12–17 years, 10.7% for those aged 5–11 years, and 7.6% for those aged 0–4 years) [5]. Residents in highly urbanized areas were more likely to receive both TCM [52] and acupuncture than those in less urbanized areas. An epidemiology survey in Taiwan also indicated that residents in areas with lower urbanization levels were less likely to use outpatient services compared to those in areas with the highest urbanization levels [53]. Interestingly, a previous US study revealed that children in families that were not poor (14.6%) were more likely to use CAM therapies than children in near-poor or poor families (9.3 and 7.0%, respectively) [5]. These findings may imply that residents living in a higher degree of urbanization with higher levels of economic development tend to receive more treatments of either western medicines or CAM.

Generally speaking, acupuncture is applied within the pediatric population not only due to its safety supported by evidence, but its benefits. Several studies such as systematic reviews have investigated the safety of acupuncture in children who belong to vulnerable groups [32, 33, 47]. In general, acupuncture has seemed to be well-tolerated in children. The adverse events from pediatric needle acupuncture were not frequently reported and mostly inconsequential, such as superficial bleeding, irritability during treatment, heat or swelling while pressing, etc. Furthermore, acupuncture treatment could customize to fit each child’s needs and relieve symptoms effectively without medication, which make acupuncture gain wider and wider acceptance. However, the precise mechanisms of acupuncture are still currently controversial [32]. The main explanation in published clinical trials is that acupuncture might conduce to a variety of neurobiological responses with the involvement of several neurotransmitters such as β endorphins, enkephalin, serotonin and so on [54]. Through those neurobiological effects, acupuncture treatment achieves therapeutic effects in many applications.

Our study had several limitations. First, based on our longitudinal cohort study, we actually followed up the same sampled pediatric enrollees from 2002 to 2011. Children who turned 18, withdrawal from the NHI and even died would drop out our study during the 10 years. Hence, the valid beneficiaries certainly decreased with year (shown in Table 1). In addition, due to protection for personal data of beneficiaries, NHIRD now is being postponed releasing for several years. Our data source seems to be the latest datasets that the NHIRD has already been released although there is still a year gap. Second, we were unable to access the detailed information of the clinic visits, such as the selected acupoints, manipulation and needle retention time. The complete and detailed information recorded in the electronic medical records of hospitals and clinics was not provided by NHIRD as part of our dataset. Third, the disease severity of patients and the treatment efficacy of acupuncture could not be estimated in this study due to the lack of information in the NHIRD. Lastly, because of the limited treatment codes of acupuncture recorded in the NHIRD, other forms of acupuncture treatment, including auricular acupuncture, scalp acupuncture and moxibustion, might not have been recorded precisely. For instance, auricular acupuncture, scalp acupuncture and moxibustion were all recorded under the same treatment codes as manual acupuncture, whereas acupressure was recorded as orthopedic manipulation. Future studies aimed to provide detailed demographic information of acupuncture treatment are warranted.

## Conclusions

In this study, we provide the latest and nationwide population-based investigation of acupuncture utilization among children in Taiwan. The prevalence of pediatric acupuncture users has substantially increased from 2002 to 2011. The major characteristics of pediatric acupuncture users included being male, greater age and a resident of a highly urbanized area and suffering from injury or disorders of the musculoskeletal system. Our findings may provide some useful information for further clinical practice, acupuncture research and even health policy decision-making.

## Data Availability

All data of this study are deposited in a properly managed public repository. We examined and analysed datasets released from the Taiwan NHIRD (http://nhird.nhri.org.tw/en/index.html), maintained and managed by National Health Research Institutes (http://www.nhri.org.tw/), Taiwan. The use of the datasets is limited to research purposes only. Applicants must follow the Computer-Processed Personal Data Protection Law (http://www.winklerpartners.com/?p=987) and related regulations of National Health Insurance Administration and National Health Research Institutes, and the agreement must be signed by the applicant and his/her supervisor upon application submission. All applications are reviewed for approval of data release.

## Abbreviations

ASD: Autism spectrum disorder
CAM: Complementary and alternative medicine
EIS: Early intervention service
ICD-9-CM: International classification of disease, 9th revision, clinical modification
LHID 2000: Longitudinal health insurance database 2000
NHI: National health insurance
NHIRD: National health insurance research database
TCM: Traditional Chinese medicine
y/o: Years old
